# To change, but not to preserve! Norm conformity following control threat only emerges for change norms but not for status quo norms

**DOI:** 10.1080/15298868.2024.2399869

**Published:** 2024-09-12

**Authors:** Janine Stollberg, Immo Fritsche, Eva Jonas

**Affiliations:** a Department of Psychology, Paris-Lodron-University Salzburg, Salzburg, Austria; b Wilhelm Wundt Institute for Psychology, University of Leipzig, Leipzig, Germany

**Keywords:** Control motivation, social change, social identity, group norms

## Abstract

Collectively pursuing social change may help people experience a sense of agency through their group when personal control is threatened, thereby restoring their sense of control. Accordingly, we proposed and found in two studies (*N* = 177 & 178) that following an experimentally manipulated threat to personal control, group members conform only to ingroup norms (vs. non-norms) framed as proposing social change, but not to those framed as preserving the status quo (in Study 1, we found this only for highly identified group members). This demonstrates the importance of collectively pursued social change for group-based control processes and qualifies the widely held belief that people reject change under conditions of threat.

When group members’ sense of personal control is threatened, they conform more strongly to what they believe the majority of other group members do and approve of (Stollberg, Fritsche, & Jonas, 2017). It has been discussed whether this reflects a general tendency to preserve the status quo under conditions of personal threat or whether threat makes people more “groupy” in the way they think and act, making social change support as likely as preservation of the status quo. It speaks for the latter account that previous studies showed threat-induced conformity to ingroup norms of *change* (in work organizations and university education). In other words: Threat to individuals’ personal control increased their support for social change when this was said to be (vs. not to be) the ingroup norm (Stollberg, Fritsche & Jonas, 2017). This contradicts the widely held notion that people become more resistant to change (“conservative shift hypothesis,” Jost et al., 2003) following threat and supports the view that they become more collective, instead.

The present paper builds on and extends this research by focusing on the unique role that support for social change may play in processes of restoring a sense of control through group membership. Extending previous theorizing, we propose that norms of social change (“we are changing the world”), in particular, are much more indicative of ingroup agency than norms of preserving the status quo (“we are keeping the world as it is”). Thus, conformity to norms of change would be much more appropriate for people to demonstrate control through their social self by thinking and acting as a group member than conformity to status-quo norms. Therefore, we proposed and tested the hypothesis that when motivated to restore control, people will support ingroup normative projects more than non-normative projects, but that this effect should be more pronounced when the ingroup normative project involves social change than when it supports the status quo.

## Motivated responses to personal control threat

Control is a fundamental human motive (Pittman & Zeigler, 2007), reflecting people’s need to perceive that they can influence important aspects of their environment through their autonomous self (Fritsche et al., 2016; Skinner, 1996; Stollberg, Fritsche, Barth, et al., 2017). When personal control seems threatened, people are motivated to restore it in primary or secondary ways (Rothbaum et al., 1982).

Primary control involves reestablishing a sense of control through the self, either by affirming personal control in another domain or by affirming collective control as a group member (i.e., “extended primary control;” Stollberg, Fritsche, Barth, et al., 2017). Specifically, group-based control theory (Fritsche, 2022; Fritsche et al., 2013) proposes group membership as a powerful resource for extended primary control: When personal control seems threatened, people turn to social ingroups to reestablish a sense of control through the (social) self (the “we” instead of the “I;” cf. social identity approach; Reicher et al., 2010; Tajfel & Turner, 1979). They do so by more strongly valuing and identifying with agentic ingroups (Agroskin & Jonas, 2013; Du et al., 2016; Fritsche et al., 2008, 2013; Greenaway et al., 2015; Proudfoot & Kay, 2018; Stollberg et al., 2015) and demonstrating collective agency by supporting ingroup thriving (Fritsche et al., 2008, 2013, 2017) and acting in accordance with salient ingroup norms (Stollberg, Fritsche, & Jonas, 2017). For example, Stollberg, Fritsche, and Jonas (2017), Study 3) asked German university students whether they would support some proposed changes in the organization of university teaching (e.g., the introduction of block teaching). Depending on the experimental condition, these proposals were either supported by a clear majority of German students compared to students from other European countries (ingroup norm condition) or for which there was a majority of foreign (but not German) students in favor of implementing the changes (no ingroup norm condition). Participants who were reminded of low (vs. high) personal control over important aspects of their lives more strongly supported those change projects that were clearly favored by their ingroup, but not those that were favored by an outgroup. Apparently, people act more as group members when their personal control is subjectively threatened. This may indicate a motivated response, as people may seek to restore a sense of control by demonstrating collective rather than personal action.

When (extended) primary control efforts seem futile, people might turn to secondary ways of restoring control, such as reducing uncertainty about how the world works and searching for order and structure (Landau et al., 2015). Such secondary control efforts in response to personal control loss were proposed and shown by compensatory control theory (Kay et al., 2008). Accordingly, threats to personal control lead people support existing systems of structure and order (e.g., social hierarchies; Friesen et al., 2014) and powerful external control agents (e.g., God and the government; Kay et al., 2008) to compensate for low personal control with external means.

Whereas compensatory control theory considers means external to the self as a resource for control perceptions (secondary control), group-based control theory considers means internal to the self (extended primary control through the social self) as a resource for control perceptions (Stollberg, Fritsche, Barth, et al., 2017). In line with a group-based control approach, extended primary control through the social self should be preferred over secondary means. In other words, when the affirmation of ingroup agency through change norm support is possible, it should be preferred over supporting the existing structure of the status quo, when personal control is at stake.

## Attitudes towards social change as a response to threatened control

Previous research found evidence for both support of social change and resistance to it when personal control was at stake. In line with compensatory control theory, it has been proposed that threat (to control) elicits a conservative shift (Jost et al., 2003), which causes people to resist and reject social change due to its unpredictable nature and as long as maintaining the status quo is still possible or social change is evitable (Friesen et al., 2019). Recent meta-analytic findings have supported the existence of a conservative shift following threat (Burke et al., 2013; Jost et al., 2017), but have also shown that this tendency can be overridden by the salience of liberal norms and group values (Burke et al., 2013; Jonas et al., 2008), allowing for a liberal shift following threat (Jost et al., 2017). Moreover, when political ideology was considered together with ingroup normative (dis)approval of social change, people’s support for change depended on ingroup norms but not ideology (Proch et al., 2019), indicating a group-based rather than ideology-based process.

## Ingroup change norm support as a means of establishing a sense of collective agency

The question, when social change support serves as a coping response for threats to personal control is not yet answered. While compensatory control theory (Landau et al., 2015) predicts an aversion to change in response to control threats, group-based control theory (Fritsche, 2022) does not. In line with group-based control, ingroup striving for social change (as opposed to preserving the status quo) may be indicative of collective agency, thereby promoting group-based control. Given that efforts to restore group-based control should be the primary or initial way in which people attempt to cope with threatened personal control, people may not oppose, but even support, social change if they view change as normative for the ingroup, since collectively pursuing change means experiencing collective agency.

According to models of agency and control (Preston & Wegner, 2005; Skinner, 1996), people can infer agency from three indicators: a) having autonomous goals, b) perceiving goal-directed action, and c) perceiving effects of the self on the environment (Fritsche, 2022; Fritsche et al., 2016). Thus, collective action should depend on whether people perceive the group to a) have an intrinsic common goal determined by the group’s internal preferences rather than external factors, b) act visibly toward the goal, and c) have discernible effects (Stollberg et al., 2015). Collectively pursuing social change goal strengthens all three of these indicators compared to supporting the status quo. First, a collective goal for change should have a stronger intrinsic relation to action and approach motivation than resistance to change has, given that the former rather represents an approach goal (approaching a novel state) and the latter rather represents an avoidance goal (avoiding the loss of the present state; McGregor et al., 2010). Approach goals, such as social change, are linked to a promotion focus and are associated with strategies of eager goal pursuit, whereas avoidance goals are associated with a prevention focus and vigilant processes (Higgins, 2012).

Second, pursuing change cannot be easily attributed to external factors, such as habit, convention, or external pressure, which are more likely to favor the status quo. Thus, attributions to intrinsic factors (e.g., collective will) should be strengthened (Kelley, 1972) when a group pursues social change rather than preserving the status quo. Relatedly, pursuing social change implicitly contrasts the ingroup to outgroups who represent the status-quo. This increases the cognitive intergroup meta-contrast (Turner et al., 1987) and, as a result, distinctiveness of the ingroup actor (Hogg, 2014). Pursuing change typically requires more effort and willpower to overcome external pressures. Also, the pursuit of change should inherently involve movement, and thus action, because it involves moving away from the status quo rather than passively remaining with it. Third, social change should lead to a novel, and therefore salient, state of the environment. Therefore, the possible outcomes of change action are more visible than the outcomes of preserving the status quo, indicating with greater certainty that an effect has occurred.

Indeed, previous research provides first evidence that people may support collective social change to regain control at the group level. Stollberg, Fritsche, and Jonas (2017) found that salient threats to personal control increased people’s conformity to ingroup norms of supporting organizational change in their work organization or changes in university teaching. Barth et al. (2018) showed that after salient threat to climate change, students more strongly conformed to norms of supporting or not supporting radical student action against sexism (a social change issue). This research indicates that group-based control restoration efforts following personal control threat are reflected in support for social change.

This contradicts the compensatory control prediction of change aversion following control threat. However, it does not address the possibility that control threatened individuals may actually prefer collective action for social change over collective action for maintaining the status quo as a means of coping with control loss. The present research fills this gap by comparing the effects of threatened personal control on people’s conformity to ingroup norms of change versus those of preserving the status quo.

## The current research

We propose that collectively pursuing social change is a powerful way for people to maintain or restore a sense of control through the (social) self when their personal control is threatened. In contrast, collectively preserving the status quo may be a more ambiguous way to demonstrate group agency and thus restore a sense of control through the group. Previous research suggests that following a threat to control, people tend to demonstrate group-based control by conforming more strongly to salient ingroup norms than to non-norms or outgroup norms (Stollberg, Fritsche, & Jonas, 2017). To test the differential value of collective social change action versus preserving the status quo, we hypothesized that conformity to ingroup norms compared to non-norms would be more pronounced following a personal control threat when the norm is social change than when it is the status quo.

We tested this prediction in two experiments with university students (Experiment 2 was pre-registered), manipulating threat to personal control, whether a proposed project was normative or non-normative for the participants’ national student ingroup (within subjects), and whether this project was framed as either social change or preservation of the status quo. We measured students’ attitudes and collective action intentions to support different proposals for how university teaching and research should be organized (e.g., allowing student mobility). While one of the proposals presented to participants represented the ingroup norm (apparently the majority of ingroup members, but the minority of outgroup members, approved of the proposal), the other two proposals represented no norm (half of ingroup and outgroup members were said to approve of the proposal). In Study 1, the normative and non-normative proposals presented to each participant were framed as either pursuing social change or maintaining the status quo (between-subjects manipulation). In Study 2, the normative and non-normative proposals presented to each participant contrasted social change with status quo framing (within-subjects manipulation). Depending on the experimental condition, when the ingroup normative proposal was framed as supporting social change, the non-normative proposals had the opposite framing as supporting the status quo, and vice versa (i.e., when the normative proposal was framed as supporting the status quo, the non-normative proposals were framed as supporting social change). Thus, Study 2 made the difference between social change and status quo proposals particularly salient by contrasting the two framings.

## Study 1

In Study 1 we tested whether conformity to ingroup norms (vs. non-norms) is most pronounced when people perceive a threat to personal control (vs. no threat) and the ingroup norm is social change (vs. preserving the status quo). We based our first study on previous research showing that following salient low (but not high) personal control people showed increased conformity with ingroup change norms compared to non-norms, or outgroup norms, of change (Stollberg, Fritsche, & Jonas, 2017). To test whether this effect is more pronounced for ingroup norms of social change than for ingroup norms of preserving the status quo, we extended the design presented by Stollberg, Fritsche, and Jonas (2017): First, we made low or high personal control salient to participants. Then we presented them with three different projects for appropriate university teaching and research, one of which was said to be supported by a clear majority of national ingroup students (82%) but only by 40–50% of national outgroup students (i.e., the normative project). The remaining two projects appeared to receive only moderate support (40–50%) from both ingroup and outgroup students (i.e., the non-normative projects).

We added an additional factor, by manipulating whether the projects presented were framed as demanding change or preserving the status quo, while holding the specific content of the proposal constant. Consistent with previous findings on group-based control, we expected increased conformity to ingroup norms compared to non-norms following a control threat as a means of demonstrating collective agency. We predicted that this effect would be stronger for ingroup norms that promoted change than for ingroup norms that preserved the status quo.

## Methods

### Participants and design

We asked 180 students on a German university campus to participate in a fictitious research project on the personal and academic living conditions of students in Central Europe. Three participants were excluded before data analysis because they did not belong to the national student ingroup (i.e., two did not study at a German university, one had studied at an outgroup university). The final sample consisted of 177 participants, 111 identified as female and 66 as male, with a mean age of *M* = 22.72 and SD = 3.45. We used a 2 personal control salience (low/high) x 2 framing (change/status quo) x 2 ingroup norm (normative/non-normative) mixed design. While personal control salience and framing were varied across participants, ingroup norm was varied within participants. Condition assignment was completely random. The study was approved by the University Ethics Board and participants gave informed consent before the experiment started.

### Procedure

#### Control salience manipulation

After a brief introduction and demographic questions, we presented the control salience manipulation, which was identical to the previous study (Stollberg, Fritsche, & Jonas, 2017). In the low control salience condition (instructions for the high control salience condition in parentheses), participants were reminded to *Take some moments to think of those aspects of your life that give you the feeling that you cannot (can) influence or (and) control important things in your life. Please, briefly jot down in your own words those two aspects of your life that make you feel most helpless (powerful)*. The control salience manipulation was followed by the German version of the PANAS (Watson et al., 1988) as a delay task.

#### Change and status quo framing

Then, participants read a brief introduction of a research project that was ostensibly conducted at universities in Poland, the Czech Republic, and Germany to gather representative information about current important university issues that European students think should be changed. We provided participants with information about three recently developed student proposals on the importance of “free, purposeless research,” “diversity of majors,” and “student mobility,” which were framed either as proposals aimed at substantial change in current practices (change framing condition) or as proposals aimed at preserving the status quo (preservation framing condition). At the end of the questionnaire, we asked participants whether they thought their ingroup of German students was satisfied with the status quo or wanted to change the current practice for each project, which served as manipulation check for social change vs. status quo framing. In the change framing condition, participants reported that their ingroup aimed at change (*M =* 4.73, *SD =* 0.94) and was not satisfied with the status quo (*M* = 3.64, *SD* = 0.90), *F*(1,173) = 45.95, *p* < .001, whereas in the status quo framing condition, participants reported that their ingroup supported the status quo (*M* = 4.89, *SD* = 1.03), and did not support social change (*M* = 3.66, *SD* = 1.04), *F*(1,173) = 60.31, *p* < .001. Thus, the framing manipulation was correctly perceived.

#### Norm salience manipulation

Normative information was manipulated within subjects by means of three proposals on different university topics. For each proposal, we provided participants with a bar chart together with four different quotes showing the average support of their national student ingroup (German students) and their national student outgroup (Polish and Czech students). This served as a norm salience manipulation. One proposal received clear normative support from the ingroup: The majority of the ingroup supported the project (82%), whereas only a few members of both outgroups were supportive (15–20%). In addition, two supportive statements were made by ingroup members, while two opposing statements were made by outgroup members. The other two propositions were non-normative, showing no clear normative support from either group: All bar graphs ranged from 40% to 50% support by ingroup and outgroup, and one positive and one negative statement were assigned to the ingroup (German students) and outgroups (Polish students, Czech students), respectively. Normative ingroup support was counterbalanced across project topics and project order. To check for successful norm manipulation, we asked participants at the end of the questionnaire whether they perceived that their ingroup of German students intended to take action on the issue. As expected, more collective action intentions were reported when the ingroup norm was supportive (*M* = 4.54, *SD* = 1.31) than when the ingroup norm was ambivalent (*M* = 4.23, *SD* = 1.08), *t*(174) = 3.10, *p* = .002.

#### Proposal support

After reading the (non)normative information for each proposal, participants indicated their support for the project. They expressed their (dis)agreement with nine items on a 7-point scale (*1 = totally disagree* to *7 = fully agree*). Their attitudes toward the project were assessed with five items: *“I think, that this project is a good thing to do,” The support of this proposal would have more advantages than disadvantages,” “To my opinion, this proposal doesn’t make much sense (reverse coded),” “I think, that this project is not important for the education at universities in the future (reverse coded),” “I support this project”, (with α’s = .82–.88, for each topic)*. Collective action intentions to support the proposal were assessed with four items: *“I would sign a petition that argues for this project,”” I would try to convince my fellow students of the benefits of this project,” “I would support the project in a public discussion,” “I would participate in a demonstration that argues for the project.” (with α’s = .91–.93, for each topic)*. We computed different mean scores for normative and nonnormative project support across the different topics, for attitudes and collective action intentions, respectively. We report our results for both dependent variables separately.

#### Ingroup identification

We measured identification with the ingroup of German students with five items on the same 7-point-scale (*1 = totally disagree to 7 = fully agree*): *“I identify myself with the group of German university students,” “Being a student of a German university has nothing to do with my identity (reverse coded).,” “Being a student of a German university is very important to me,” “I recognize myself in the group of German university students,” “I do not feel any bond with the group of German university students (reverse coded).,”* with *M* = 4.64, *SD =* 1.41, and α = .87. Upon completing the experiment, participants were debriefed, thanked, and received a chocolate bar.

## Results

To test our hypothesis that conformity to ingroup norms vs. non-norms following personal control threat is more pronounced when the ingroup norm demands social change than when it supports the status quo, we conducted a 2 personal control salience (low/high) x 2 framing (change/status quo) x 2 ingroup norm (normative/non-normative) mixed ANOVA with repeated measurement on the last factor. We ran separate analyses for the dependent variables attitudes toward the proposal and collective action intentions to support the proposal and report the results together.

The results showed a main effect of framing on attitudes toward the proposal, *F*(1,172) = 9.07, *p* = .003, η^2^ = .05, and on collective action intentions, *F*(1,172) = 4.92, *p* = .028, η^2^ = .03: proposals that were framed as preservation of the status quo were more supported, attitudes 95% CI [5.31, 5.65], collective action intentions 95% CI [4.38, 4.84] than projects that were framed as demanding change, attitudes 95% CI [4.93, 5.28], collective action intentions 95% CI [4.00, 4.48], independent of control salience and ingroup norm salience, as all multivariate interaction effects were not significant, control salience x (non) normative support, *F*(1,172)_attitudes_ = 0.04, *p* = .837, η^2^ < .001 and *F*(1,172)_intentions_ = 0.13, *p* = .721, η^2^ = .001, framing x (non) normative support, *F*(1,172)_attitudes_ = 1.14, *p* = .287, η^2^ = .01 and *F*(1,172)_intentions_ = 0.48, *p* = .490, η^2^ = .003, and control salience x framing x (non) normative support, *F*(1,172)_attitudes_ = 0.17, *p* = .681, η^2^ = .001 and *F*(1,172)_intentions_ = 0.15, *p* = .702, η^2^ = .001. There were also no further between subjects effects, for control salience, *F*(1,172)_attitudes_ = 0.002, *p* = .965, η^2^ < .001 and *F*(1,172)_intentions_ = 1.12, *p* = .292, η^2^ = .01, or control salience x framing, *F*(1,172)_attitudes_ = 0.42, *p* = .520, η^2^ = .002 and *F*(1,172)_intentions_ = 0.39, *p* = .534, η^2^ = .002. For a comprehensive overview, the descriptive values for attitudes and collective action intentions are available in the supplementary material.

### Secondary analysis: moderation by ingroup identification

As the assumed group-based control process of increased conformity to ingroup norms following a control threat, should be more pronounced in those who identify strongly with the salient ingroup of German students, we conducted a secondary analysis, considering ingroup identification as an additional moderator. We used *R*, version 4.4.0 (R Core Team, 2024) and the packages *reshape*, version 0.8.9 (Wickham, 2022) and *interactions*, version 1.1.5 (Long, 2022) to run a mixed linear regression model to test whether the predicted control salience x framing interaction on ingroup (non-)normative support depended on participants identification with their ingroup. After including ingroup identification as an additional predictor and all possible two-, and three-way interactions on ingroup norm support, the personal control salience x framing x ingroup norm x identification interaction turned out significant, for collective action intentions, *b* = 0.96, *t*(174) = 2.18, *p* = .030, and attitudes, *b* = 0.84, *t*(174) = 2.47, *p* = .014, respectively.

**Figure 1.**
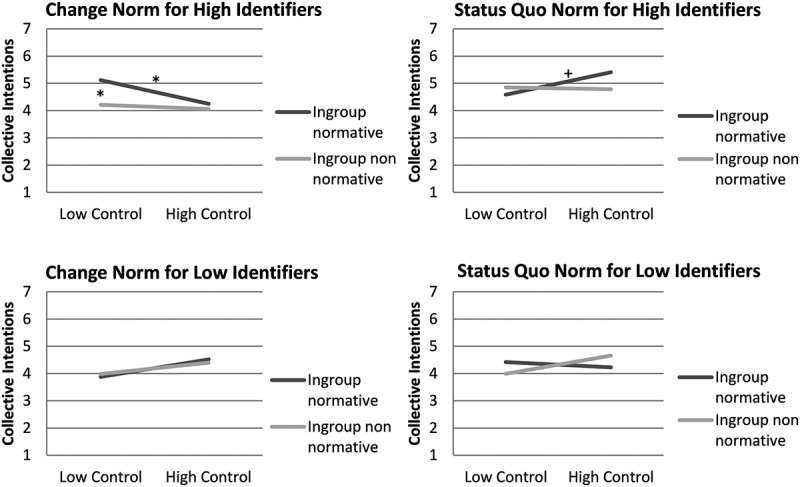
Ingroup identification and norm conformity following control threat: differences in ingroup normative and nonnormative support between social change and status quo framing conditions.

Note. The figure shows differences in norm conformity among highly identified ingroup members as a function of personal control salience and norm framing (change vs. status quo). Significant differences between normative and non-normative collective action intentions, as well as differences between control salience conditions, are indicated by *p < .05, and marginal significant differences by ^+^*p* < .10.

Looking at the difference between normative project support and non-normative project support for the different combinations of conditions, revealed significant effects only for highly identified ingroup members that were in the low control salience and change framing condition (see Figure 1): Highly identified ingroup members conformed more to ingroup norms than to non-norms, when they perceived low personal control and when the norm was framed as supporting change, significant for collective action intentions, *b* = 0.92, *t*(165) = 2.02, *p* = .045, and marginal significant for attitudes, *b* = 0.65, *t*(165) = 1.83, *p* = .069. In contrast, highly identified ingroup members showed no difference in support for ingroup norms compared to non-norms, when personal control was low and the norm was framed as supporting the status quo, for collective action intentions, *b* = −0.26, *t*(172) = −0.66, *p* = .510, or for attitudes, *b* = −0.42, *t*(172) = −1.38, *p* = .169. This provides preliminary support for the group-based control prediction that only when participants were motivated to restore control (but not when personal control was affirmed) they did support ingroup normative change projects more than ingroup non-normative change projects (but made no difference between normative and non-normative status quo projects).

Furthermore, we also looked at the simple effect of personal control salience on change versus status quo norm support for high identifiers. Personal control threat had a positive effect on ingroup norm support when the norms were framed as supporting social change: Highly identified German students showed increased support for ingroup change norms when personal control was low compared to when it was high, significant for collective action intentions, *b* = 0.88, *t*(165) = 1.99, *p* = .048, and marginal significant for attitudes, *b* = 0.62, *t*(165) = 1.82, *p* = .071. Unexpectedly, when the norm was framed as preserving the status quo, personal control threat had a negative effect on ingroup norm support: Highly identified German students showed less conformity to status quo norms when personal control was low than when it was high, marginal significant for collective action intentions, *b* = −0.83, *t*(172) = −1.95, *p =* .052, and significant for attitudes, *b* = −0.85, *t*(172) = −2.64, *p =* .009.

## Discussion study 1

The results provide first conditional evidence that joining collective efforts for change is a means for people to maintain a sense of control through the (social) self when personal control has been thwarted. Specifically, we found the predicted ingroup change norm effect following personal control threat only if ingroup identification was considered as additional moderator. People who were highly identified with their ingroup of German university students supported ingroup normative project proposals for social change more than non-normative proposals after their personal control was threatened (but not when it was not threatened). Most importantly, this effect occurred only when these proposals demanded social change, but not when they advocated the status quo. This is in line with group-based control theory (Fritsche, 2022), that threatened individuals can restore subjective control by demonstrating ingroup agency by acting as a group member, i.e., conforming to salient ingroup norms (Stollberg, Fritsche, & Jonas, 2017). What is new is that conforming to norms of social change seems to be a much more attractive means for people to regain control than conforming to norms of preserving the status quo. As we argue, this should be because collective change goals are more clearly indicative of collective agency than status quo goals.

However, these results should be interpreted with caution and considered preliminary, as contrary to our initial hypothesis, we found the predicted interaction effect on norm support only for people who identified strongly with their group. Although the study design should have made ingroup membership salient to some degree for all participants, only high identifiers may have paid sufficient attention to the group-based opportunities to restore control by supporting collective change.

In addition, including ingroup identification as additional moderator lowered the statistical power of the analysis. While the moderation by ingroup identification is consistent with group-based control theory, as restoring a sense of control through the self by demonstrating *group* agency requires that people view the group as a representative of their self (Fritsche et al., 2013), it requires further testing in a pre-registered study with more statistical power. Thus, we set up Study 2, where we contrasted change vs. status quo framing within participants. This allowed for more statistical power, and at the same time it should strengthen the collective norm framing.

## Study 2

Study 2 was a pre-registered experiment that we set up as a conceptual replication of Study 1. We used the basic design of Study 1 but strengthened the manipulation of framing and increased the statistical power. We did this by first contrasting ingroup change projects with status quo projects within participants. Specifically, each participant was presented with both types of projects, with the ingroup normative project framed in either change or status quo terms and the two non-normative projects framed in opposite terms. Second, we refined the wording of the ingroup member’s statements indicating change vs. status quo projects. Consistent with the results of Study 1, we predicted that following a threat to personal control, individuals show more conformity to ingroup norms relative to non-norms when the ingroup norm represents social change than when it represents preserving the status quo. The study was approved by the University Ethics Board and participants gave informed consent before the experiment began.

We pre-registered our hypotheses and analysis plan for Study 2, which are available at https://aspredicted.org/g6k2s.pdf.

## Methods

### Participants and design

One hundred and eighty-one German university students participated in an experiment on the same fictitious research project on the personal and academic living conditions of students in Central Europe as used in the previous study. We used a 2 personal control salience (low/high) x 2 framing (change/status quo) x 2 ingroup norm (normative/non-normative) mixed design. Personal control salience was manipulated between subjects, while framing and ingroup norm were manipulated within subjects. Assignment to conditions was completely random. Sample size was determined by an a priori power analysis to detect the predicted three-way interaction, with a 5% probability of error, 80% statistical power, and an estimated correlation among repeated measure of *r* = .02, for an effect size of *f* = .17 (estimated from previous norm x control salience interaction effects), resulting in a required sample of approximately *N* = 160. After excluding two participants according to our pre-registered exclusion criteria (i.e., they indicated not to belong to the national ingroup of university students), and one, who quitted the experiment, the final sample consisted of 178 participants. One hundred and eight identified themselves as female, 69 as male, and one did not respond; the mean age of the participants was *M* = 24.16, with *SD* = 4.33.

### Procedure

#### Control salience manipulation

After a brief introduction and demographic questions, participants read the same control salience manipulation, making high vs. low personal control salient, as in Study 1. Again, the control salience manipulation was followed by the German version of the PANAS (Watson et al., 1988) as a delay task.

#### Change and status quo framing for ingroup (non-)norms

We used the same norm salience manipulation with bar charts accompanied by group member statements, as in Study 1. Participants first read a short introduction about a research project that was supposedly conducted at universities in Poland, the Czech Republic, and Germany to gather representative information about current important university issues (“free research without purpose,” “diversity of majors,” and “student mobility”) that European students think should be changed. All participants received information about one normative proposal that was supported by their ingroup (82% of German students supported it) and about two non-normative proposals that were not clearly supported by either the ingroup or the outgroup (40–50% of German, Polish and Czech students supported them). The normative proposal was always presented as the second issue, while the first and third issues were non-normative proposals. When the normative proposal called for change, the non-normative proposals supported the status quo, and vice versa. Project topics were counterbalanced across conditions. The manipulation of normative change and non-normative status quo change norms (and vice versa) is available in the supplementary Material.

#### Norm manipulation check

At the end of the norm proposition questionnaire, we asked participants whether they thought their ingroup of German students was satisfied with the status quo or whether their ingroup wanted to change current practice for the normative and nonnormative topics. This served as a manipulation check of change vs. status quo norm framing. When the ingroup norm was framed as a change norm, participants reported that their ingroup wanted change, (*M* = 5.05, *SD* = 1.45) and was not satisfied with the status quo, (*M* = 2.90, *SD* = 1.41), *F*(1.173) = 51.70,*p* < .001, whereas when the ingroup norm was framed as the status quo norm, participants reported more satisfaction with the status quo for their ingroup (*M* = 4.55, *SD* = 1.58), and less collective intention to change (*M* = 3.57, *SD* = 1.64), *F*(1,173) = 10.81, *p* = .001. Thus, we deemed our norm framing manipulation to be successful.

#### Proposal support

After reading the normative and nonnormative proposals, participants indicated their support for each proposal. They responded to nine items on a 7-point-scale (1 = *totally disagree* to 7 = *fully agree*), adopted from Study 1 that assessed their attitudes toward the project, α = .84–.88 (for each topic), and collective action intentions to support the project, α = .86–.92 (for each topic). Again, we calculated different mean scores for support of the ingroup normative project and the nonnormative projects, which served as dependent variables, separately for attitudes and collective action intentions.

### Ingroup identification

We measured identification with the ingroup of German students with six items adopted from Leach et al. (2008): *“I feel a bond with students of German universities,” “I am glad to be a student of a German university,” “The fact that I am a student of a German university is an important part of my identity,” “Being a student of a German university gives me a good feeling,” “I feel solidarity with students of German universities,” “Being a student of a German university is an important part of how I see myself.,”* with *M* = 4.15, *SD* = 1.36, α = .87. Upon completing the experiment, participants were debriefed, thanked, and received a chocolate bar.^1^

## Results

**Figure 2.**
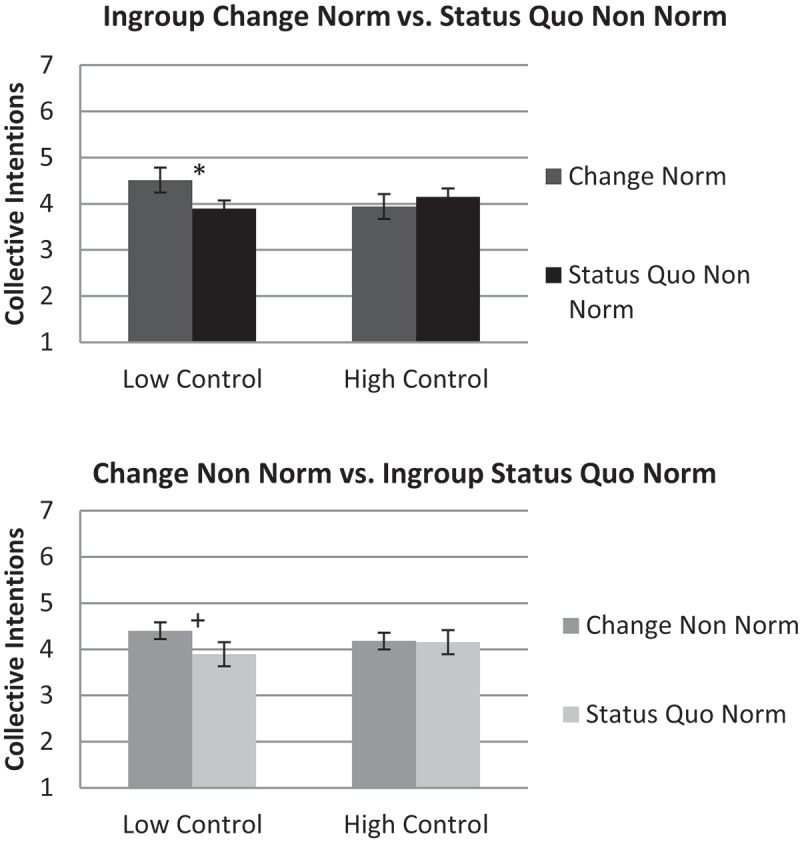
Norm conformity following control threat: contrasting change with the status quo within participants.

To test whether participants with low perceptions of personal control show increased conformity to ingroup change norms, we conducted a 2 personal control salience (low/high) x 2 framing (change vs. status quo) x 2 ingroup norm (normative/non-normative) ANOVA with repeated measurement on the last two factors. The results showed the predicted multivariate three-way interaction of personal control salience x framing x ingroup norm, significant for collective action intentions, *F*(1,174) = 4.65, *p* = .033, ƞ^2^ = .03, and marginal for attitudes, *F*(1,174) = 3.62, *p* = .059, ƞ^2^ = .02 (see Figure 2). For a comprehensive overview, the descriptive values for attitudes and collective action intentions are available in the supplementary material.

The figure shows differences for normative and non-normative collective action intentions, comparing change norms with status quo non norms (and vice versa) within participants, depending on control salience conditions. Significant differences are indicated by *p < .05, and marginal significant differences by ^+^*p* < .10. Error bars indicate standard errors.

When personal control was at stake, participants’ support for ingroup normative and non-normative projects depended on norm framing: Participants low in personal control, who were presented with an ingroup change norm, reported more collective action intentions to support that norm, *M* = 4.51, *SD* = 1.89, than to support the nonnormative status quo projects, *M* = 3.89, *SD* = 1.26, *F*(1,174) = 4.47, *p* = .036, ƞ^2^ = .03. A similar, but non-significant, pattern occurred for attitudes (change ingroup norm: *M =*5.27, *SD* = 1.46 vs. status quo non-norm: *M* = 4.94, *SD* = 1.06), *F*(1,174) = 1.65, *p* = .200, ƞ^2^ = .01.

In contrast, as a marginally significant trend, participants low in personal control, who were presented with an ingroup status quo norm reported less collective action intentions to support that norm, *M* = 3.89, *SD* = 1.85, than to support non-normative change projects, *M* = 4.40, *SD* = 1.07, *F*(1,174) = 3.10, *p* = .080, ƞ^2^ = .02 (for attitudes: status quo ingroup norm: *M* = 4.97, *SD* = 1.59 compared to change non- norm: *M* = 5.46, *SD* = 0.90, *F*(1,174) = 3.56, *p* = .061, ƞ^2^ = .02).

There were no differences between ingroup normative and nonnormative support, when high personal control was salient, for ingroup change norm, *F*(1,174)_intentions_ = 0.47, *p* = .496, ƞ^2^ = .003, *F*(1,174)_attitudes_ = 1.08, *p* = .300, ƞ^2^ = .01, for ingroup status quo norm, *F*(1,174)_intentions_ = 0.06, *p* = .812, ƞ^2^ < .001, *F*(1,174)_attitudes_ = 0.17, *p* = .683, ƞ^2^ = .001.

Looking at the simple effect of personal control salience, participants reported descriptively more support for ingroup change norms when personal control was low (for collective action intentions: *M* = 4.51, *SD* = 1.89, for attitudes: *M* = 5.27, *SD* = 1.46), than when it was high (for collective action intentions: *M* = 3.94, *SD* = 1.63, for attitudes: *M* = 4.94, *SD* = 1.06), but the expected simple effect of control salience on support for ingroup change norms was not significant for either collective action intentions, *F*(1,174) = 2.28, *p* = .133, ƞ^2^ = .01, or attitudes *F*(1,174) = 0.65, *p* = .422, ƞ^2^ = .004. In sum, the results indicate that conformity to ingroup norms following a threat to personal control becomes attractive when the norm demands social change, but not when it supports the status quo.

### Secondary analysis: moderation by ingroup identification

We conducted a secondary analysis to test for moderated moderation by ingroup identification. Using *R*, version 4.4.0 (R Core Team, 2024) and the packages *reshape*, version 0.8.9 (Wickham, 2022) and *interactions*, version 1.1.5 (Long, 2022), we ran a mixed linear regression model to test whether the predicted control salience x framing x ingroup norm interaction depended on participants’ identification with their ingroup. After including ingroup identification as an additional predictor and all possible two- and three-way interactions on ingroup normative support, the results showed the multivariate three-way interaction of personal control salience x change framing x ingroup norm for collective action intentions, *b* = 1.29, *t*(176) = 1.99, *p* = .048, and marginal significant for attitudes, *b* = 0.95, *t*(176) = 1.77, *p* = .078. However, ingroup identification did not moderate the personal control x change framing x ingroup norm interaction effect, as the interaction of personal control salience x change framing x ingroup norm x identification was not significant, for collective action intentions, *b* = −0.05, *t*(176) = −0.10, *p* = .921, and attitudes, *b* = −0.17, *t*(176) = −0.41, *p* = .682, respectively.

## Discussion study 2

The results of Study 2 confirm our previous findings that following a threat to personal control, ingroup members support projects that are normative for their ingroup more than non-normative projects, but only when the ingroup project is about social change and not when it is about preserving the status quo. This supports the group-based control notion that people respond to threats to personal control with increased group-based cognition and behavior when these promise to demonstrate collective agency, which should be possible when collectively pursuing change but less so when aiming to preserve the status quo.

Interestingly, as a marginally significant trend, control threatened participants supported the ingroup normative project less than the non-normative project when the ingroup normative project was about preserving the status quo, whereas the non-normative projects represented social change. This might invite speculation about a strong version of the group-based control hypothesis of collective change: Perhaps status quo norms are not only less indicative of ingroup agency than change norms but may actually hinder the impression of collective agency and thus the possibility of restoring control through the social self. This may be particularly the case when people are simultaneously able to pursue change projects, thereby highlighting the agentic ambiguity of status quo projects.

As a further interpretation, people whose personal control was threatened may have distorted their perception of how normative the change projects were for their ingroup. Given that in our study about 50% of ingroup members were still reported to approve of the non-normative projects, control-motivated participants may have interpreted this as an indication of a moderately strong ingroup norm, which they then followed. Similar effects have been observed for individuals experiencing self-uncertainty, who interpreted ambivalent group norms in an uncertainty-reducing manner (Smith et al., 2007). Even more, threatened participants may have aimed to contribute to social change becoming the future norm of their ingroup (see loyal deviance, Packer & Miners, 2014) as an indirect route to ingroup agency.

## General discussion

When their personal control is threatened, people may reestablish a sense of control through their collective self. Specifically, they may engage in behaviors that are normative for their group, indicating group-based action and thus the agency of people’s social selves. We hypothesized that collectively pursuing social change is an important component of restoring group-based control (Fritsche, 2022) because it is a much more explicit and visible demonstration of collective agency than supporting the status quo. Thus, after experiencing a threat to personal control, people should be more inclined to follow ingroup norms of social change than ingroup norms of preserving the status quo. We tested this hypothesis in two experiments.

In support of our basic hypothesis, Studies 1 and 2 showed that people who were reminded of low personal control (but not those who were reminded of high personal control) were more inclined to behave in accordance with a perceived ingroup norm compared to a non-norm when the ingroup norm represented social change, but not when it represented the status quo. Whereas in Study 1 this pattern was true only for highly identified but not for low identified group members, in Study 2 it was true for all participants. The more pronounced effects for high identifiers are consistent with the argument of group-based control theory that demonstrating collective agency only restores control through the social self when people consider the group to be representative of their self (i.e., when they are identified with the group). However, they require further replication, as ingroup identification did not moderate the effects in Study 2. As the experimental procedure of comparing German with Czech and Polish university students should have made the ingroup identity of German university students salient to some extent for all participants, we expected control threat to increase conformity to ingroup change norms for all participants. This was supported by the findings of Study 2. The fact that high levels of self-reported ingroup identification were not necessary to show the predicted pattern in Study 2 might go back to a strengthened change vs. status-quo framing manipulation. In Study 1, participants were presented with three propositions (one ingroup normative and two non-normative propositions), all of which were similarly framed as either supportive of change or supportive of the status quo. In Study 2, however, the ingroup normative proposal was always framed opposite to the two non-normative proposals, thus contrasting change vs. status quo proposals within participants. This gave us the opportunity to test whether participants preferred to support change over preserving the status quo. At the same time, it should have increased the salience of the change vs. status quo nature of the ingroup normative proposal, thus strengthening the framing manipulation. This may explain why the framing effect was present for all threatened participants in Study 2, but only for high identifiers in Study 1.

### Norm conformity as a response to threat

The present studies support previous findings that threat increases norm conformity (Schindler et al., 2022; Stollberg, Fritsche, & Jonas, 2017). However, they significantly extend this research by explaining these effects in terms of striving for group agency and by highlighting an important boundary condition. According to our theorizing and findings, people conform more to ingroup norms than to non-norms in response to threat only when norm conformity indicates collective agency (i.e., social change). Specifically, in the present studies, threats to personal control only increased conformity to ingroup norms of social change (in Study 1 only for high identifiers), but not to those of preserving the status quo. However, given that in Study 2 the simple effect of threat of control on support for ingroup change norms occurred only as a nonsignificant trend, these results should be considered preliminary and require replication.

We observed that conformity to ingroup change norms following threat was most pronounced for indicators of behavioral support, but weaker and sometimes only marginally significant for attitudinal support. Although we had no prior hypotheses about this, one might speculate that for control-deprived individuals, taking action to support a group norm is of particular value in experiencing and visibly demonstrating agency through their (collective) self, as opposed to simply agreeing with a given norm. This possibility should be taken into account in future studies.

### Status quo support as a motivated response to control threat

In contrast to the group-based control perspective, previous theorizing has emphasized that threat to control may cause collective inaction and aversion to (social) change (Landau et al., 2015). From this compensatory control perspective (Landau et al., 2015), people rely on the status quo and support established structures, such as existing hierarchies (Friesen et al., 2014; but see; Lautenbacher & Fritsche, 2023) and governmental systems (e.g., Kay et al., 2008), when their personal control is at stake, in order to make the world structured and predictable, which prevents them from supporting change unless it is inevitable (Friesen et al., 2019). The present findings show that a personal need for control can also be positively associated with support for social change when that change is linked to group goals and collective engagement toward it. Thereby, they speak for the primacy of group-based control (extended primary control; Stollberg, Fritsche, Barth, et al., 2017) over secondary compensatory control efforts when both are available under conditions of personal control threat. Future research could investigate whether these processes are limited to personal threats as opposed to system-level threats (van der Toorn et al., 2017).

### Limitations

#### Moderation by ingroup identification and statistical power

As a limitation, the interpretation of ingroup identification as a moderator of ingroup change norm effects following control threat is limited by the low statistical power of the analysis in Study 1. Including identification as an additional moderator reduced the statistical power to detect the proposed interaction effect. Future studies should use larger samples to test whether ingroup change norm support of control threatened individuals is more pronounced among highly identified ingroup members, or whether mere salience of group membership is sufficient.

#### Comparing control threat effects to control salience

As a further limitation, the manipulations of personal control threat did not include a neutral control condition, but only contrasted salient low vs. high personal control. Thus, technically, the effects could be explained in terms of salient high control rather than low control. However, such an explanation seems rather unlikely: First, only when people were reminded of low personal control but not high personal control, they differed in their conformity to change vs. status-quo ingroup norms. An alternative account in terms of salient high control would imply that people, by default, like change norms more than status-quo norms. And it would have to explain why this difference is eliminated under high control. Perhaps the affirmation of personal control eliminates group-based control defenses that people would otherwise need to protect themselves from latent control threats through fluctuating daily reminders of lacking personal control. However, this is speculative and would support the basic argument of group-based control theory. Second, previous research on control threat effects that included a neutral control condition found an increase in group-based responses when low control was salient. For example, in different series of studies by Fritsche et al. (2008), 2013; Du et al., 2016) framing death as uncontrolled increased people’s group-based cognition and motivation (e.g., ingroup identification, ingroup support) compared to both a controlled death treatment and a neutral control group (i.e., thinking about dental pain). In a similar vein, Agroskin and Jonas (2013) found increased derogation of outgroup protesters for people who were asked to interpret a short poem that was either about death or lack of control, compared to a poem about winter (i.e., neutral control condition; indirect effects). Nevertheless, although it seems unlikely that the control manipulation effects can be explained merely in terms of salient high control, future studies on control-threat induced change norm conformity should include a neutral control condition.

## Conclusion

Social change can be ambivalent in times of personal threat and societal crisis. It may increase uncertainty in people who desire the world to be a structured and orderly place in the face of crises that can trigger perceptions of chaos and randomness. People may then reject social change as a compensatory control response (Jost et al., 2003; Kay et al., 2008). At the same time, however, as the present results show, people support social change through their ingroup as a means of restoring their sense of control through the (social) self under conditions of personal threat (Fritsche, 2022; Stollberg, Fritsche, & Jonas, 2017). This increased willingness of control-deprived group members to collectively pursue social change may even lay the groundwork for actual individual empowerment and concerted collective action to resolve societal crises. These group-based control responses of pursuing collective change may offer some hope for whether and how humanity can solve its existential crises of today and the days to come, such as climate catastrophe, species extinction, or the denial of human rights.

## Supplementary Material

Supplementary material_revised.docx

## Note

1. In line with our preregistration, we also measured perceived ingroup agency as additional variable for exploratory purpose.
