# Supplementary material for: To change, but not to preserve! Norm conformity following control threat only emerges for change norms but not for status quo norms
Source: Self Identity. 2024 Sep 12;23(5-6):484–504. doi: 10.1080/15298868.2024.2399869 (PMC11441394; doi:10.1080/15298868.2024.2399869)

**Supplementary Material**

**To change, but not to preserve!**

**Norm conformity following control threat only emerges for change norms but not for status quo norms**

Content:

- Descriptive values for attitudes and collective action intentions for Study 1 (Table 1)
- Descriptive values for attitudes and collective action intentions for Study 2 (Table 2)
- Study Material: Examples of norm salience manipulation for Study 2, translated to English

**Tables 1 and 2**

*Descriptive values for normative and nonnormative support depending on control threat and change vs. status quo framing for Studies 1 and 2*

|  |  | | Study 1 | | | |
| --- | --- | --- | --- | --- | --- | --- |
|  | Norm manipulation | | Ingroup Norm | Non Norm | Ingroup Norm | Non Norm |
|  |  | | Change | Change | Status Quo | Status Quo |
| Low Control | *Attitudes* | *M*  *(SD)* | 5.29  (1.20) | 4.99  (0.87) | 5.42  (1.19) | 5.45  (0.91) |
|  | *Collective action intentions* | *M*  *(SD)* | 4.39  (1.66) | 4.02  (0.99) | 4.50  (1.56) | 4.44  (1.21) |
| High Control | *Attitudes* | *M*  *(SD)* | 5.15  (1.41) | 4.99  (0.91) | 5.53  (1.40) | 5.52  (0.81) |
|  | *Collective action intentions* | *M*  *(SD)* | 4.36  (1.80) | 4.20  (1.18) | 4.79  (1.77) | 4.72  (1.13) |

|  |  | | Study 2 | | | |
| --- | --- | --- | --- | --- | --- | --- |
|  | Norm manipulation | | Ingroup Norm | Non Norm | Ingroup Norm | Non Norm |
|  |  | | Change | Status Quo | Status Quo | Change |
| Low Control | *Attitudes* | *M*  *(SD)* | 5.27  (1.46) | 4.94  (1.06) | 4.97a+  (1.59) | 5.46a+  (0.90) |
|  | *Collective action intentions* | *M*  *(SD)* | 4.51a*  (1.89) | 3.89a*  (1.26) | 3.89a+  (1.85) | 4.40a+  (1.07) |
| High Control | *Attitudes* | *M*  *(SD)* | 5.01  (1.50) | 5.29  (0.92) | 4.93  (1.32) | 5.04  (0.94) |
|  | *Collective action intentions* | *M*  *(SD)* | 3.94  (1.63) | 4.15  (1.29) | 4.11  (1.62) | 4.18  (1.26) |

*Note.* Significant differences within participants are marked for each combination of conditions, with ** = p* < .05, and marginal significant differences with + *= p <* .10*.*

**Study Material: Norm Salience Manipulation Study 2**

The examples show the original norms salience manipulation used in Study 2, translated to English. For anonymization reasons, the exact university affiliation of students’ quotes is disguised and replaced by “Ingroup University” or “Outgroup University”, respectively. The original anonymized material for all versions is available at OSF (link provided in main manuscript).

**Example for Change Norm vs. Status Quo Non Norm**

Outgroup University

Outgroup University

Ingroup University

Ingroup University

Ingroup University

Outgroup University

Outgroup University

Ingroup University

**Example for Status Quo Norm vs. Change Non Norm**

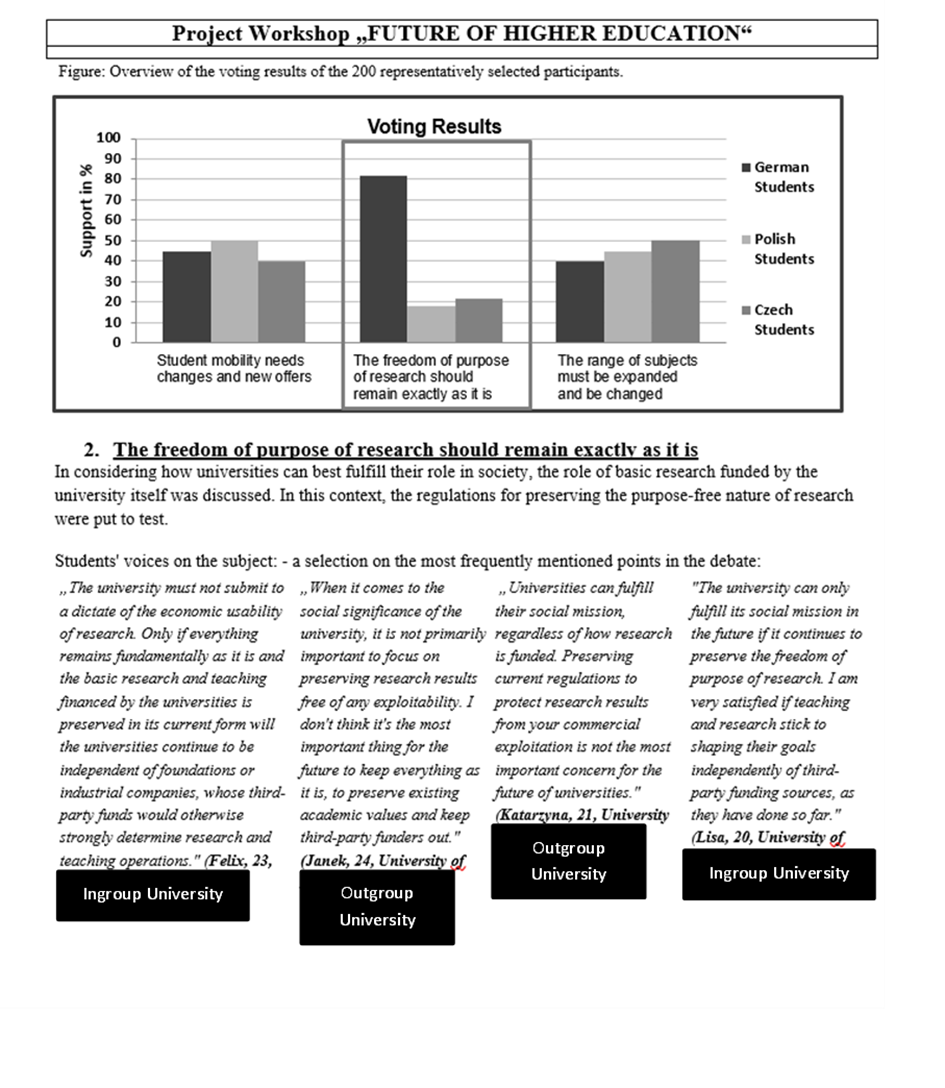

Supplement: Supplementary material_revised.docx [file PSAI_A_2399869_SM9227.docx]
